# Establishment of a primed pluripotent epiblast stem cell in FGF4-based conditions

**DOI:** 10.1038/srep07477

**Published:** 2014-12-17

**Authors:** Jin Young Joo, Hyun Woo Choi, Min Jung Kim, Holm Zaehres, Natalia Tapia, Martin Stehling, Koo Sung Jung, Jeong Tae, Hans R. Schöler

**Affiliations:** 1 Department of Cell and Developmental Biology, Max Planck Institute for Molecular Biomedicine, Röntgenstrasse 20, 48149 Münster, Germany; 2 Department of Animal Biotechnology, College of Animal Bioscience and Technology, Konkuk University, Seoul 143-701, Republic of Korea; 3 Infertility Clinic Center, Haesung Hospital, Chun An 331-950, Republic of Korea

## Abstract

Several mouse pluripotent stem cell types have been established either from mouse blastocysts and epiblasts. Among these, embryonic stem cells (ESCs) are considered to represent a “naïve”, epiblast stem cells (EpiSCs) a “primed” pluripotent state. Although EpiSCs form derivatives of all three germ layers during *in* *vitro* differentiation, they rarely incorporate into the inner cell mass of blastocysts and rarely contribute to chimera formation following blastocyst injection. Here we successfully established homogeneous population of EpiSC lines with efficient chimera-forming capability using a medium containing fibroblast growth factor (FGF)-4. The expression levels of *Rex1* and *Nanog* was very low although *Oct4* level is comparable to ESCs. EpiSCs also expressed higher levels of epiblast markers, such as *Cer1*, *Eomes*, *Fgf5*, *Sox17*, and *T,* and further showed complete DNA methylation of *Stella* and *Dppa5* promoters. However, the EpiSCs were clustered separately from E3 and T9 EpiSC lines and showed a completely different global gene expression pattern to ESCs. Furthermore, the EpiSCs were able to differentiate into all three germ layers *in vitro* and efficiently formed teratomas and chimeric embryos (21.4%) without germ-line contribution.

During the early developmental stages, pluripotent cells can be derived from the inner cell mass (ICM) and post-implantation epiblast cells. The pluripotent embryonic stem cells (ESCs) are derived from the ICM of blastocyst-stage embryos *in* *vitro,* whereas the pluripotent cells derived from post-implantation stage epiblasts, termed epiblast stem cells (EpiSCs) represent a more differentiated state than pluripotent ESCs. Therefore, EpiSCs have been referred to as “primed” pluripotent cells in order to distinguish them from the more potent “naïve” pluripotent ESCs^1^. However, EpiSCs do not efficiently contribute to the embryonic tissue after blastocyst injection, the efficiency being approximately 0.5% (2/385)^1^,^2^. Further, EpiSCs and naïve pluripotent stem cells differ in colony morphology^1^, global gene expression patterns, including micro RNAs^3^, X chromosome inactivation pattern^4^, and signaling pathways required for self-renewal^5^. Although mouse and human ESCs are both derived from the preimplantation embryos, human ESCs^1^,^6^ are distinct from mouse ESCs and share more common characteristics with EpiSCs with respect to their critical dependence on basic fibroblast growth factor (bFGF) and Activin/Nodal signaling for pluripotency and self-renewal^2^,^7^.

Self-renewal and maintenance of pluripotency in EpiSCs depends upon the extrinsic stimulation of Activin/Nodal pathway along with a parallel induction of bFGF signaling^2^. However, Activin/Nodal and bFGF do not support the generation of a pure population of EpiSCs; mixed populations were found to co-exist in a single culture dish^1^,^8^. In the mixed cell population, only Oct4-green fluorescence protein (GFP)-positive EpiSCs readily contributed to chimeras while GFP-negative EpiSCs did not, as they represented the late epiblast cells^3^,^8^. Taken together, these findings suggest that the Activin/Nodal signaling pathway might support the existence of two distinct cell populations. Therefore, we speculated that a homogenous population of EpiSCs could be maintained in presence of a factor supporting only the *Oct4-GFP*-positive cell population. So we attempt to characterize growth factors important for self-renewal in homogenous population of EpiSCs.

In the present study, we cultured EpiSCs in FGF4-supplemented medium in the absence of Activin and bFGF. FGF4 was proposed as an autocrine self-renewal promoting factor in human ESCs^9^. Feldman et al. also suggested that *Fgf4* gene was important for supporting proliferation of ICM of mouse blastocyst and postimplantation embryos^10^. *Oct4* and *Sox2* regulate the expression of their target gene, *FGF4*^4^,^11^, which is restrictedly expressed in the developing embryos and is believed to play a key role in embryonic survival and patterning^2^,^5^,^12^.

The results of the present study demonstrated that EpiSCs could self-renew in the presence of FGF4 without the exogenous stimulation of the Activin/Nodal signal. Therefore, our study suggests that FGF4 is an important factor for self-renewal and maintenance of homogenous EpiSC populations that express epiblast markers and efficiently form chimeras.

## Results

### Generation of a homogenous population of EpiSCs from embryonic day 5.5 (E5.5) and 6.5 (E6.5) epiblasts

Conventional EpiSC medium results in a heterologous population of EpiSCs^8^. In the present study, we used a transgene where GFP expression was controlled by Oct4 regulatory elements. The transgene represents a 10 kbp genomic fragment (genomic *Oct4* fragment 18 kbp: GOF18) and contains crucial regulatory elements including a promoter and two upstream enhancers, called the distal enhancer (DE) and proximal enhancer (PE)^13^. Mouse EpiSCs containing the GOF18 GFP transgene express GFP because of the PE, whereas ESCs express GFP because of the DE. The conventional EpiSC medium containing Activin and bFGF does not result in a homogenous Oct4-positive EpiSC population^8^. To test if we could obtain a homogenous Oct4-positive EpiSC population, we employed a culture medium containing FGF4, which was replaced for Activin and bFGF. FGF4 was proposed as a factor capable of enhancing the self-renewal of human ESCs^9^. *Fgf4*, a target gene of Oct4 and Sox2, support the proliferation of ICM of mouse blastocyst and postimplantation embryos (E5.5 and 6.5)^10^. *Fgf4*^-/-^ embryos degenerated shortly after uterine implantation, and *Fgf4*^-/-^ blastocysts showed severely impaired proliferation of the ICM, which was completely rescued by adding recombinant FGF4 protein in the medium^10^. FGF4, together with LIF and steel factor, promotes the maintenance of pluripotency of embryonic germ cells (EGCs)^14^.

**Figure 1:**
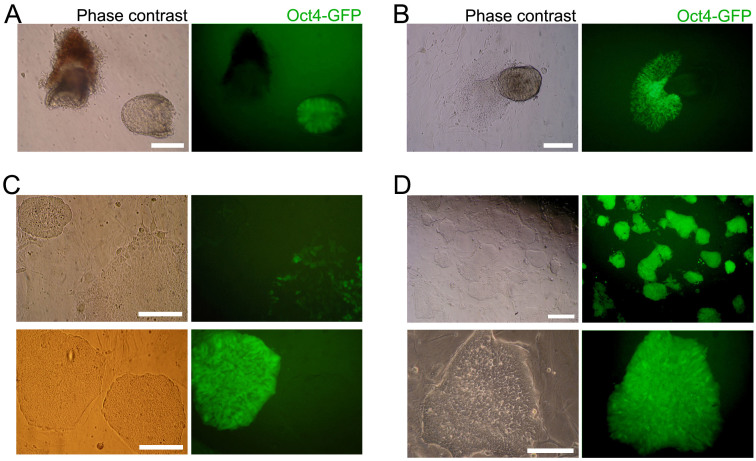
Generation of EpiSCs from E6.5 epiblast. (A) E6.5 embryos were recovered from GOF18 transgenic mice. GFP-positive epiblasts were used for generating EpiSCs. (B) GFP-positive outgrowths spreading out from the epiblasts. (C) Until the third passage, only a few cells and colonies in the culture were GFP-positive. (D) Pure populations of GFP-positive cells were established after three rounds of FACS. Scale bars are 100 μm.

E5.5 and 6.5 embryos were recovered from GOF18 transgenic mice. GFP-positive epiblasts were dissected to separate the embryo/epiblast from the extraembryonic ectoderm (Fig. 1A and Supplementary material Fig. S1A). GFP-positive epiblasts were cultured in FGF4-supplemented medium (F4 medium). GFP-positive outgrowths spreading out from epiblasts were disaggregated and plated onto gelatin-coated dish containing F4 medium (Fig. 1B). GFP-positive EpiSCs were established after subsequent selection and culture. Until the third passage, most of the cells in the culture were GFP-negative and only a few cells and colonies were GFP-positive (Fig. 1C). However, a pure population of EpiSCs expressing GFP could be established only by repeated (triplicate) fluorescence-activated cell sorting (FACS) purification at each passage (Fig. 1D and Supplementary material Fig. S1B). These EpiSCs, designated as F4-EpiSCs, were found to maintain homogenous GFP-positive colonies maintaining normal karyotype even after a long-term propagation (over 100 passages for about 6 months) (Supplementary material Fig. S1C). When the F4-EpiSCs were transferred into conventional EpiSC medium containing Activin and bFGF, GFP-negative cells appeared again and became heterogeneous (data not shown). These results indicate that F4 medium is supportive for the maintenance of homogenous Oct4-GFP-positive populations of EpiSCs. Of the two F4-EpiSC lines derived from E5.5 and E6.5 epiblasts, F4-EpiSCs from E6.5 epiblast were further analyzed.

### F4-EpiSCs represent a primed pluripotent state

**Figure 2:**
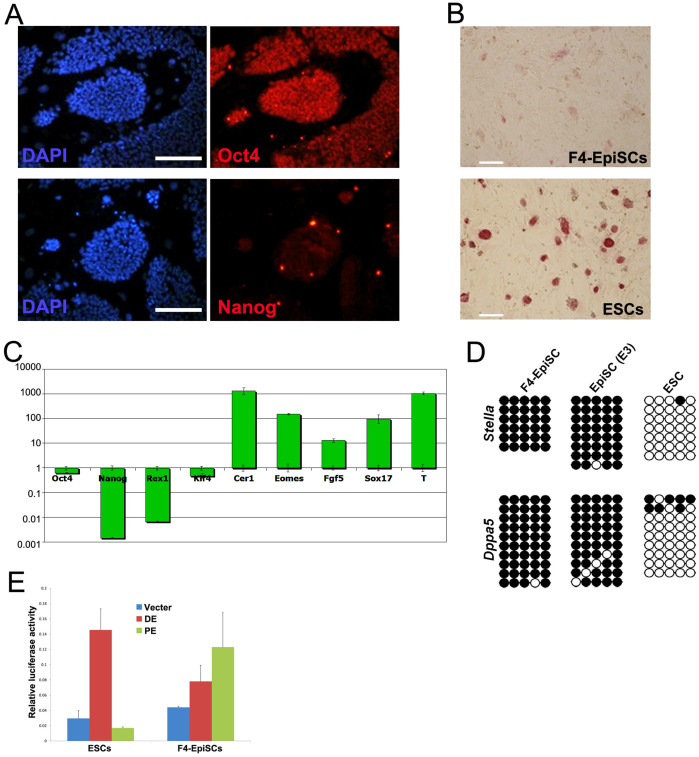
Primed pluripotency—a unique feature of F4-EpiSCs. (A) The GFP-positive F4-EpiSCs were stained positive for Oct4, but were weakly stained by anti-Nanog antibody. (B) F4-EpiSCs showed a very weak alkaline phosphatase activity, thus demonstrating their primed pluripotent state. (C) Representative real-time RT-PCR analysis data of F4-EpiSCs (from 3 lines of single-cell clones) and control ESCs using mouse ESC-specific and EpiSC markers. (D) DNA methylation patterns of the promoter regions of *Stella* and *Dppa5*. (E) Analysis of the *Oct4* enhancer activity in ESCs and F4-EpiSCs. Relative luciferase activity was normalized to the activity of the empty vector. Scale bars are 100 μm.

The GFP-positive F4-EpiSCs were positive for Oct4, but barely expressed Nanog as determined by immunofluorescence staining (Fig. 2A). When compared to ESCs, F4-EpiSCs were very weakly positive for alkaline phosphatase activity (Fig. 2B). We generated 3 lines of single-cell clones from F4-EpiSCs, which were subjected to real-time RT-PCR analysis. Real-time RT-PCR analysis revealed that the expression levels of mouse ESC-specific markers, *Nanog* and *Rex1*, were much lower in F4-EpiSCs when compared to those in ESCs (about 900- and 100-fold, respectively) (Fig. 2C). Our findings demonstrate that F4-EpiSCs are distinct from ESCs and are consistent with previous studies that demonstrated *Oct4* expression both in naïve as well as primed pluripotent stem cells, whereas *Nanog* expression levels were significantly lower in EpiSCs (Fig. 2C)^3^,^15^,^16^.

Notably, F4-EpiSCs expressed higher levels of epiblast markers, such as *Cer1*, *Eomes*, *Fgf5*, *Sox17*, and *T*, when compared to ESCs (Fig. 2C). Both *Stella* and *Dppa5* (markers for germ cells and naïve pluripotent stem cells) were unmethylated in ESCs, but hypermethylated in EpiSCs (Fig. 2D). As expected, the promoter regions of *Stella* and *Dppa5*, which were unmethylated in mouse ESCs, were hypermethylated in F4-EpiSCs. In addition, luciferase assay showed that F4-EpiSCs utilized epiblast-specific PE of *Oct4* (Fig. 2E). Collectively, these data show that F4-EpiSCs are distinct from ESCs and represent a primed pluripotent state, which can be maintained in the presence of FGF4 without an exogenous stimulation of the Activin/Nodal pathway.

### F4-EpiSCs can be distinguished from other EpiSC lines

**Figure 3:**
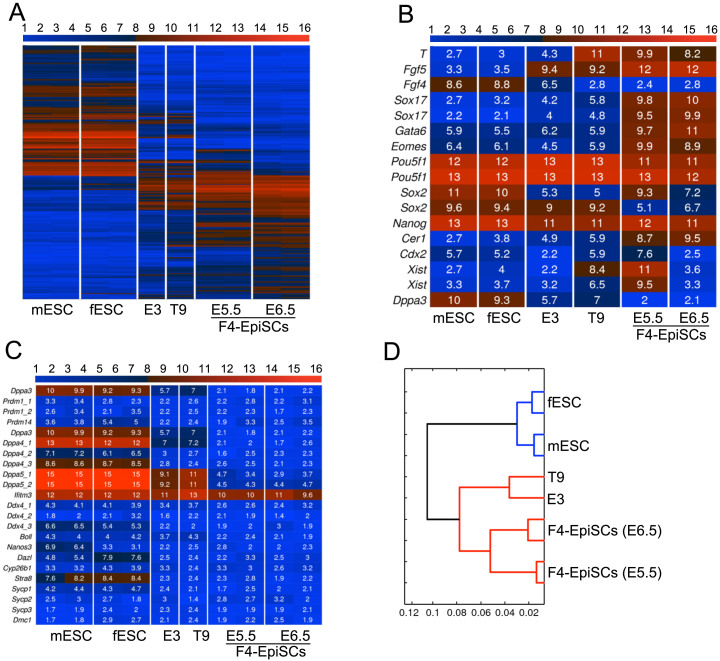
Gene expression profiles of F4-EpiSCs, ESCs, and other EpiSC lines. (A) Heat map of the global gene expression profiles of ESCs, E3, T9 EpiSC lineS, and F4-EpiSCs. mESC and fESC denote, male ESC and female ESC, respectively. (B) Heat map of pluripotency- and EpiSC-related genes. (C) Heat map of germ cell and meiosis markers. (D) Hierarchical clustering shows that two F4-EpiSC lines clustered close together, but distinct from other EpiSC lines (E3 and T9).

We next investigated whether F4-EpiSCs (derived from E5.5 and E6.5) showed similar characteristics to other well-described EpiSC lines, such as the T9 and E3 cell lines^5^,^12^. F4-EpiSCs were found to be distinct from other EpiSCs lines (Fig. 3A) and expressed more EpiSC markers when compared to E3 and T9 lines (Fig. 3B). Similar to a previous observation in other EpiSC lines, F4-EpiSCs did not express germ cell and meiosis markers, except *lfitm3* (*Fragilis*)^17^ (Fig. 3C). Therefore, F4-EpiSCs are most similar to the epiblast in their characteristics, and may represent epiblast cells *in* *vivo*, excluding germ cell precursors. Strikingly, F4-EpiSCs showed gene expression patterns that were distinctive compared to other EpiSC lines (Fig. 3D and Supplementary material Fig. S2). Therefore, our study suggests that F4-EpiSCs may be a novel cell type and could be easily distinguished from other EpiSC lines reported previously.

### Efficient chimera formation ability of F4-EpiSCs

**Figure 4:**
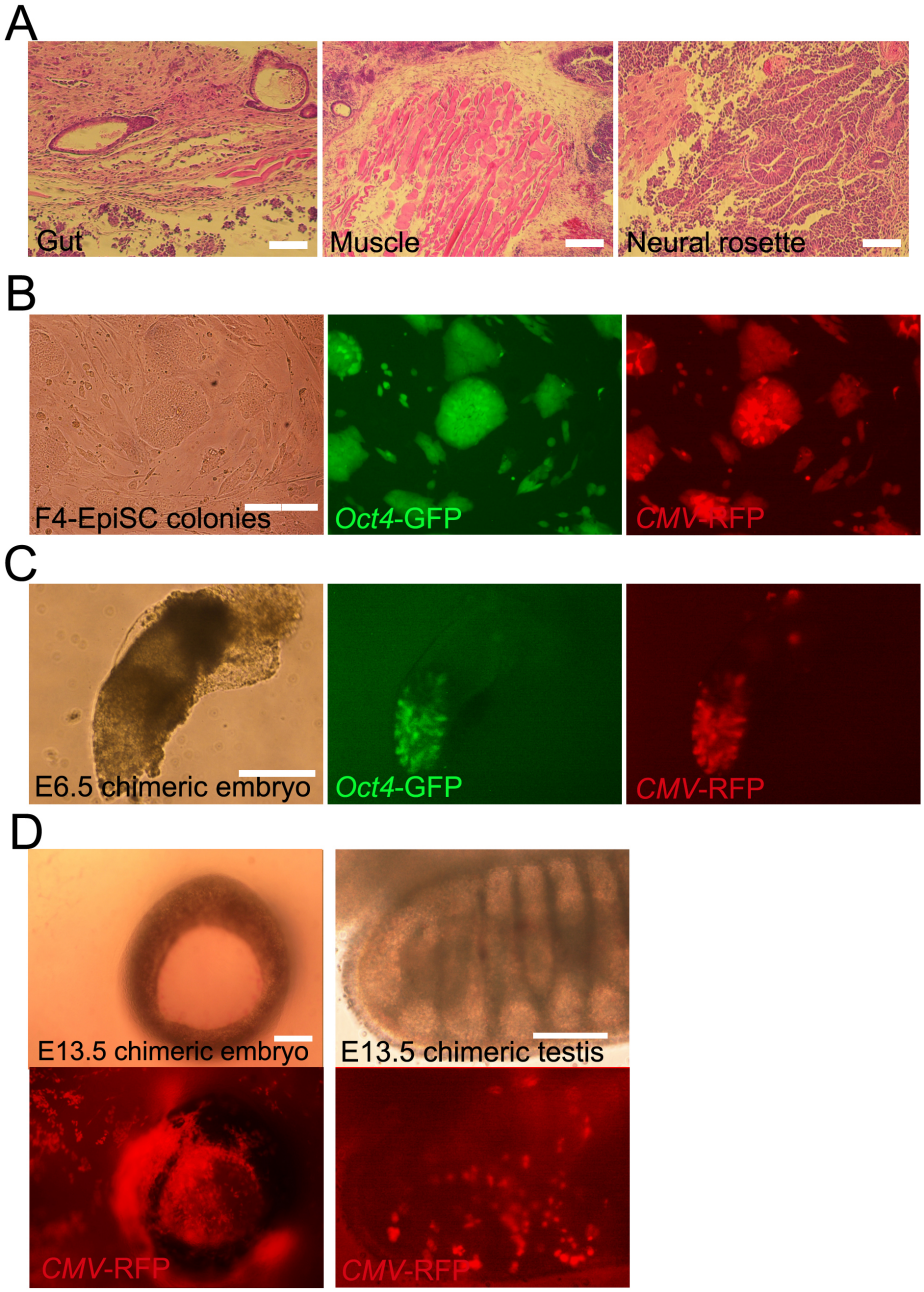
Differentiation potential of F4-EpiSCs *in* *vivo*. (A) Hematoxylin and eosin staining of teratomas derived from F4-EpiSCs. (B) F4-EpiSCS infected with lentivirus constitutively expressing the *red fluorescence protein* (*RFP*) transgene, expressed both RFP and GFP. (C) E6.5 chimeric embryos showing GFP and RFP dual-positive epiblasts and the low contribution to extraembryonic tissues (only RFP-positive). (D) Representative images of the chimeric embryos. E13.5 chimeric embryos, in which RFP-positive cells were detected in the face around an eye (left panel) and testis (right panel). Oct4-GFP was not detected in gonadal tissue in any of the six chimeric embryos. Scale bars are 100 μm.

The observation that F4-EpiSCs resembled epiblast cells, which could differentiate to form all three germ layers during gastrulation, led us to investigate whether F4-EpiSCs showed a better differentiation potential than other EpiSC lines (which could hardly form chimeras). We therefore tested the multi-lineage differentiation potential of F4-EpiSCs (derived from E6.5) *in vivo*. First, F4-EpiSCs were transplanted subcutaneously into the dorsal flanks of immunodeficient (SCID) mice to assess their teratoma forming ability. It was observed that after 3 months of injection, teratomas containing gut, muscle, and neural tissues were formed (Fig. 4A). Next, we assessed the ability of F4-EpiSCs to form chimeras by injecting these cells into mouse blastocysts. Previous reports have suggested that EpiSCs, which represent primed pluripotent state, rarely if at all form chimera^12^,^18^. Chimera forming ability is, therefore, regarded as one of the hallmarks of “naïve” pluripotency^19^. However, we speculated that F4-EpiSCs might have a higher potential when compared to E3 and T9 EpiSC lines, since homogenous populations of F4-EpiSCs maintain Oct4-GFP over continuous subcultures and display more epiblast-like characteristics. To monitor the presence of F4-EpiSCs in chimeric embryos, we infected F4-EpiSCs with a lentivirus constitutively expressing the red fluorescence protein (RFP). The F4-EpiSCs, which harbored *Oct4*-*GFP* and *CMV*-*RFP*, expressed GFP and showed a ubiquitous expression of RFP in the presence of an active *Oct4* (Fig. 4B). Initially, we observed chimerism in an early developmental stage, E6.5 embryo. Chimeric E6.5 embryos showed a high contribution of F4-EpiSCs in epiblast (both GFP- and RFP-positive) and a low contribution in extraembryonic tissues (only RFP-positive) (Fig. 4C). Chimerism could also be observed in E13.5 embryos (Fig. 4D). Notably, F4-EpiSCs contributed to somatic germ layers, including tissues of the testis, but not to the germ cell lineage, as Oct4-GFP was not active in RFP-positive cells. The efficiency of somatic chimerism, which showed RFP-positive cells in embryos, was approximately 21.4% (6/28). Thus, F4-EpiSCs exhibited a higher differentiation potential when compared to other previously reported EpiSC lines that could hardly contribute to chimerism^2^. These data indicate that F4-EpiSCs contribute to all three germ layers efficiently, except germ cells. This difference could be attributed to the low expression levels of *Nanog* in F4-EpiSCs. Nanog-deficient ESCs could self-renew and contribute to all three germ layer tissues in chimeras, but fail to contribute to germ cells. Therefore, Nanog is specifically required for germ cell formation^20^. Exclusive contribution to somatic lineage of F4-EpiSCs is the distinctive feature from other EpiSCs lines, including GFP-positive E3 cell line^8^.

## Discussion

In the present study, we demonstrated that primed pluripotent EpiSCs have a higher differentiation potential than previously thought; when a homogenous population is propagated under suitable culture conditions. Further, we showed that homogenous populations of EpiSCs, which contribute significantly to chimeric embryos, could be maintained in FGF4-supplemented culture medium. In addition, F4-EpiSCs displayed distinctive gene expression patterns compared to other EpiSC lines (E3 and T9 lines). Notably, the F4-EpiSCs population seems to be somatic epiblast lineage that has the ability to differentiate into derivatives of all three somatic lineages, but not the germline, as they lacked both the expression of the germ cell markers and the contribution to germ cell lineage in chimera.

We derived F4-EpiSCs from E5.5 and 6.5 GOF18 transgenic embryos. E5.5 F4-EpiSCs were derived from developmentally earlier stage than E6.5 F4-EpiSCs, but the global gene expression pattern was very similar to E6.5 F4-EpiSCs. Striking differences were the levels of *Cdx2* and *Xist* expression (Fig. 3B). E5.5 F4-EpiSCs express more trophoblast marker, *Cdx2*, than E6.5 F4-EpiSCs, although the expression level of other trophoblast marker *Eomes* was not different. Most of all, E5.5 EpiSCs were female cell line and E6.5 EpiSCs were male cell line. Thus there is no X chromosome inactivation in E6.5 F4-EpiSCs. On the other hand, *Xist* expression level of E5.5 F4-EpiSCs was very high (Fig 4B), indicating that inactive X chromosome is present as in somatic cells. Therefore, F4-EpiSCs should have the same X chromosome state as somatic cells; one of the two X chromosomes is inactive and the other X chromosome is active, otherwise two X chromosomes are all active if they are in a naïve pluripotent state. As the E6.5 F4-EpiSCs were male cells (XY), X chromosome in E6.5 F4-EpiSCs is always active. However, active X chromosome (Xa) of naïve pluripotent stem cells can be distinguishable from that of somatic cells. *Xist* gene region 1 (-381 to +74) is partially methylated in Xa of naïve pluripotent cells, but completely methylated in Xa of somatic cells^21^. Bisulfite DNA sequencing analysis showed that *Xist* gene region 1 of E6.5 F4-EpiSCs was completely methylated as in EpiSCs cultured in conventional EpiSC medium, indicating that Xa of F4-EpiSCs is not in naïve, but in primed pluripotent state (Supplementary material Fig. S3). Epiblasts around E6.5 comprise two different types of epiblast cells: primordial germ cell (PGC) precursors and somatic cell precursors that contribute to all the three germ layers. Various studies have reported that EpiSCs express germ cell markers. Germ cell marker expression is a characteristic of ESCs, and thus raises the possibility that the origin of ESCs could be germ cells^22^. However, our findings revealed that F4-EpiSCs did not express the germ cell markers, thus significantly differing from ESCs and other EpiSC lines. Taken together, these data indicate that the established F4-EpiSC line does not harbor the PGC precursor population, and thus represents a pure population of EpiSCs. Our results are further strengthened by previous observations that germ cell marker expressing EpiSCs represent a heterogeneous cell population, therefore could be mixed with naïve ESC-like cells. Hence, it is likely that the mixed ESC-like cells contribute to chimera after blastocyst injection. Considering this, a pure population of EpiSCs should not express germ cell markers and exclusively represent somatic epiblast cell population. Therefore, our study established that the population of EpiSCs comprises pure homogenous somatic stem cells.

Chimera forming ability has been regarded as a marker of “naïve” pluripotency^19^. However, the results of our study proved that primed pluripotent F4-EpiSCs were capable of generating chimera efficiently (without germline contribution) after blastocyst injection. Therefore, it is the germline contribution, and not the chimera forming ability, that represents the unique property of naïve pluripotent cells. Although Hayashi et al. advocated the germline contributing ability of a few cells in an EpiSCs culture, the cells retaining germline contribution could be naïve pluripotent cells in a heterogeneous cell population under a given culture condition^23^. Indeed, ESCs easily lose the ability to contribute to the germline in chimeras during culture^22^. Therefore, primed pluripotent cells reflect a less diverse differentiation potential (lack the germline competency) compared to naïve pluripotent cells.

Naïve pluripotency could be re-established in EpiSCs either by exposure to leukemia inhibitory factor (LIF), or by overexpressing pluripotency-related genes such as *Klf4*, *Nr5a2*, *Nanog*, or *c*-*Myc*^15^,^18^,^24^,^25^,^26^. In addition, induced EpiSCs (iEpiSCs) could be generated by using the same factors that were employed for iPSC generation, by just changing the culture medium that supports the differentiation of EpiSCs^8^. Therefore, stem cells retain some degree of plasticity, thus maintaining their identity, which in turn depends upon the signaling factors required for self-renewal, but not the origin of cells. On the other hand, a recent study by Di Stefano et al. reported FGF-dependent iPSCs, which display naïve ESC-like properties under EpiSC culture conditions^27^. They suggested that the naïve pluripotent state of iPSCs could be induced regardless of the growth factors present in culture. Collectively, these findings indicate that both culture condition as well as cell origin are important for establishing cellular identity.

In conclusion, in the present study, we successfully maintained pure homogenous populations of EpiSCs in FGF4-based culture medium. The F4-EpiSCs population exclusively comprises cells that possess the ability to differentiate into derivatives of all three somatic lineages, but not the germline. Therefore, the novel EpiSC culture system established in the present study may goal in a better understanding of the innate properties related to primed pluripotent stem cells.

## Methods

The methods were carried out in accordance with the approved guidelines and all experimental protocols were approved by the Institutional Animal Care and Use Committee of the Max-Planck Institute for Molecular Biomedicine and Konkuk University.

### Mice and Cell Culture

All mouse strains were either bred and housed at the mouse facility of the Max Planck Institute (MPI), Germany, or bought from Harlan or Jackson laboratories, CA, USA. Animal handling was in accordance with the MPI animal protection guidelines and the German animal protection laws. The derivation and characterization of GOF18 EpiSCs is described elsewhere^5^. In brief, E6.5 embryos (129/Sv female X C57BL6 and DBA/2 background GOF18^+/+^ male) were collected and transferred into mouse embryonic fibroblast (MEF) medium. For dissection, deciduas were removed with needle, and the extraembryonic ectoderm was separated from the epiblast by using hand-pulled glass pipettes. After washing with PBS, the epiblast was cultured on inactivated MEFs in FGF4-based medium^28^ with some modification : DMEM (GIBCO BRL Grand Island, NY, USA) containing 20% FBS, 2 mM glutamine, nonessential amino acids, and 25 ng/ml FGF4. EpiSC medium^2^: DMEM/F12 (GIBCO BRL) containing 20% knockout serum replacement (GIBCO BRL), 2 mM glutamine, nonessential amino acids, Activin A and 5 ng/ml bFGF. After initial culture on MEFs for three to five passages, EpiSC colonies were spilt and transferred onto dishes that had been pre-coated with gelatin for 30 min. For conditioning medium, irradiated MEFs were seeded at a density of 5 × 10^4^ cells/cm^2^ and incubated in MEF medium for 96 h. The conditioned medium was filtered with a 0.45 µm filter, and FGF4 (25 ng/ml) or bFGF (5 ng/ml) was added. Cell clumps were replated on gelatin-coated dishes, and the medium was changed every 48 h. Animals were maintained and used for experimentation under the guidelines of the Institutional Animal Care and Use Committee of the Max-Planck Institute for Molecular Biomedicine and Konkuk University.

### Blastocyst Injection

Blastocysts were collected from B6C3F1 mice. F4-EpiSCs were recovered by treatment with 0.25% trypsin EDTA, and placed in a drop of PBS under mineral oil. The blastocysts were placed in an adjacent drop of PBS containing 10% fetal bovine serum (v/v). Both GFP and RFP positive cells (n = 10–15) were loaded into an injection pipette and injected into B6C3F1 blastocysts by Piezo Micromanipulator (Prime-tech Ltd, Tsuchiura, Ibaragi, Japan). Each of the 8–15 injected blastocysts were transferred into the uteri of pseudopregnant ICR mice.

### DNA Methylation Analysis

Genomic DNA was isolated from ESCs and EpiSCs. Subsequently, the DNA samples were subjected to bisulfite treatments using an EpiTect Bisulfite Kit (QIAGEN) following the manufacturer's protocol. PCR amplification of the promoter regions of *Stella* and *Dppa5* was performed by employ the following primer sets and annealing temperatures:

*Stella* 1^st^ sense 5′-TTTTTTTATTTTGTGATTAGGGTTG-3′;

*Stella* 1^st^ antisense 5′-CTTCACCTAAACTACACCTTTAAAC-3′ (161 bp, 45°C);

*Stella* 2^nd^ sense 5′-TTTGTTTTAGTTTTTTTGGAATTGG-3′;

*Stella* 2^nd^ antisense 5′-CTTCACCTAAACTACACCTTTAAAC-3′ (116 bp, 55°C);

*Dppa5* 1^st^ sense 5′-GGTTTGTTTTAGTTTTTTTAGGGGTATA-3′;

*Dppa5* 1^st^ antisense 5′-CCACAACTCCAAATTCAAAAAAT-3′ (140 bp, 45°C);

*Dppa5* 2^nd^ sense 5′-TTTAGTTTTTTTAGGGGTATAGTTTG-3′;

*Dppa5* 2^nd^ antisense 5′-CTTCACCTAAACTACACCTTTAAAC-3′ (132 bp, 55°C).

The PCR products were then subcloned into the pGEM-T Easy vector (Promega, Madison, WI), and were sequenced. The BiQ Analyzer software (Max Planck Society, Germany) was employed for the visualization and quantification of the bisulfite sequence data.

### Flow Cytometry

For FACS sorting, hybrid cells were dissociated with 0.25% trypsin EDTA, neutralized with DMEM supplemented with 10% FCS, washed with PBS, and then filtered through a 40-mm nylon mesh to remove large cell clusters. The cells were resuspended in appropriate culture medium and aliquots of 5 × 10^6^ cells/ml were transferred into polystyrene tubes. Subsequently, they were analyzed using FACSAria™ III cell sorter (BD Biosciences). Highly intense GFP-positive cells were isolated by fluorescence-activated cell sorting (FACS) using FACSDiVa™ software (Becton Dickinson, San Jose, CA).

### Immunocytochemistry

For immunocytochemistry, F4-EpiSCs were fixed with 4% paraformaldehyde for 20 min at room temperature. After washing with PBS, cells were treated with PBS containing 10% normal goat serum and 0.03% Triton X-100 for 45 min at room temperature. Following incubation with primary antibodies: anti-Nanog (Cosmo Bio, REC-RCAB0002PF, 1:500) and anti-Oct4 (Abcam, ab19857, 1:500) overnight at 4°C, the cells were washed with PBS and then visualized after a 3-h incubation with appropriate fluorophore-labeled secondary antibodies (Alexa Fluor 488 or 568; Molecular Probes, Eugene, OR, USA) according to the manufacturer's instructions. Specimens were analyzed using an Olympus Fluorescence microscope and images were acquired with a Zeiss Axiocam camera.

### Luciferase Assay

For quantifying the relative Oct4 enhancer activity of F4-EpiSCs, an Oct4 upstream sequence ~2 kb (Oct4-2 kb) containing distal enhancer (DE) and proximal enhancer (PE), and either ΔDE, or ΔPE was cloned into pGL3 basic vector (Promega, USA). The Oct4 upstream sequence (~2 kb) containing DE and PE was derived from pOct4-GFP plasmid, which was digested and ligated to the *Kpn*I/*Bgl*II sites of the pGL3 basic vector.

The pGL3-Oct4/ΔDE or pGL3-Oct4/ΔPE reporter constructs were prepared in two steps. First, a fragment of DE 5′or PE 5′ was PCR-amplified from pOct4-GFP plasmid using specific primer pairs, digested with *Kpn*I and *Mlu*I restriction enzymes and cloned into pGL3 basic vector to obtain pGL3-DE 5′ or pGL3-PE 5′ plasmids, respectively. Subsequently, a fragment of DE 3′ or PE 3′ was PCR amplified from pOct4-GFP plasmid using primer pairs carrying *Mlu*I and *Bgl*II restriction sites, respectively. The amplified fragment was digested and ligated to *Mlu*I/*Bgl*II sites of either pGL3-DE 5′or pGL3-PE 5′ plasmids. The primers used were as follows: Oct4-up-5-KpnI ATATATGGTACCCTAGTTCTAAGAAGACTTGGGACTTCAGAC, Oct4-ATG-3′-BglII- ATATATAGATCTGGGGAAGGTGGGCACCCCGAGCCGGGGGCCT, DE del-3-MluI- ATATATACGCGTCCTGTCTGTATTCAATACCAACCT, DE del-5-MluI- ATATATACGCGTTCCTAGCCCTTCCTTAATCTGCTA, PE del-3-MluI- ATATATACGCGTCCCATTACTGGCCTGGTGCTTAGT, PE del-5-MluI- ATATATACGCGTCAGATATTTCTTCTCTCTACCCAC.

Luciferase assays were performed in MEF, ESCs, and F4-EpiSCs using the Dual-Luciferase Reporter Assay System (Promega, USA). For the reporter assay of Oct4 enhancer activity, pGL3-Oct4/ΔDE, or pGL3-Oct4/ΔPE vectors (for firefly luciferase activity) and pRL-TK vector (for *Renilla* luciferase activity) were transfected individually into MEF, ESCs, and F4-EpiSCs. After 48 h of transfection, growth medium was removed and cells were rinsed in 1× PBS. Subsequently, the cells were lysed using 1× passive lysis buffer (PLB) and incubated for 10 min at room temperature with shaking. The cell lysate was then transferred to 1.5 ml new tube and centrifuged at 10, 000 rpm for 5 min at 4°C. Ten microliters of the supernatant was transferred to a 96-well plate and then analyzed for luciferase expression by luminometry. Each experiment was performed in triplicate and the values obtained were recorded as relative light units (RLU).

### Microarray Analysis

DNA-free RNA samples to be analyzed by microarrays were prepared using Qiagen RNeasy columns with on-column DNA digestion. Three hundred nanograms of total RNA per sample were used as input for the linear amplification protocol (Ambion, Austin, TX, http://www.ambion.com), which involved synthesis of T7-linked double-stranded cDNA and 12 h of *in vitro* transcription, incorporating biotin-labeled nucleotides. Purified and labeled cRNA was then hybridized for 18 h onto MouseRef-8 v2 gene expression BeadChips (Illumina, Inc., San Diego, http://www.illumina.com) following the manufacturer's instructions. After washing, the chips were stained with streptavidin-Cy3 (GE Healthcare, Chalfont St. Giles, UK, http://www.gehealthcare.com) and scanned using the iScan reader (Illumina, Inc., San Diego, USA) and accompanying software. Samples were exclusively hybridized as biological replicates.

The intensities for each bead were mapped to gene information using BeadStudio 3.2 (Illumina). Background correction was performed using the Affymetrix Robust Multi-array Analysis (RMA) background correction model, variance stabilization was performed using the log_2_ scaling, and gene expression normalization was calculated with the quantile method implemented in the lumi package of R-Bioconductor. Data post-processing and graphics were performed with in-house developed functions in Matlab.

## Author Contributions

J.Y.J., J.T.D. and H.R.S. wrote the main manuscript text and design the concept of the experiment. J.Y.J., H.W.C., M.J.K., H.Z., N.T. and M.S. performed experiment and assembled data. K.S.J. performed data analysis. All authors reviewed the manuscript

## Supplementary Material

Supplementary Information Dataset 1
