# Supplementary material for: Establishment of a primed pluripotent epiblast stem cell in FGF4-based conditions
Source: Sci Rep. 2014 Dec 17;4:7477. doi: 10.1038/srep07477 (PMC4268649; doi:10.1038/srep07477)
Supplement: Supplementary Information — Dataset 1 [file srep07477-s1.doc]

**Establishment of a primed epiblast stem cell in FGF4-based conditions**

**Jin Young Joo^1,2,3^, Hyun Woo Choi^2^, Min Jung Kim^2^, Holm Zaehres^1^, Natalia Tapia^1^, Martin Stehling^1^, Koo Sung Jung^3^, Jeong Tae Do^2,*^ and Hans R. Schöler^1,*^**

^1^Department of Cell and Developmental Biology, Max Planck Institute for Molecular Biomedicine, Röntgenstrasse 20, 48149 Münster, Germany

^2^Department of Animal Biotechnology, College of Animal Bioscience and Technology, Konkuk University, Seoul 143-701, Republic of Korea

^3^Infertility Clinic Center, Haesung Hospital, Chun An 331-950, Republic of Korea

**^*^**Corresponding authors:

Jeong Tae Do: E-mail: dojt@konkuk.ac.kr

Tel: +82-2-450-3673,

Fax: +82-2-455-1044

Hans R. Schöler: E-mail: office@mpi-muenster.mpg.de

Tel: +49-251-70365-300,

Fax: +49-251-70365-399

**Key words: Naïve pluripotency, primed pluripotency, epiblast stem cells, fibroblast growth factor-4, chimeric embryo.**

**Running title: Somatic lineage-specific epiblast stem cells**

**Supplementary information**

**
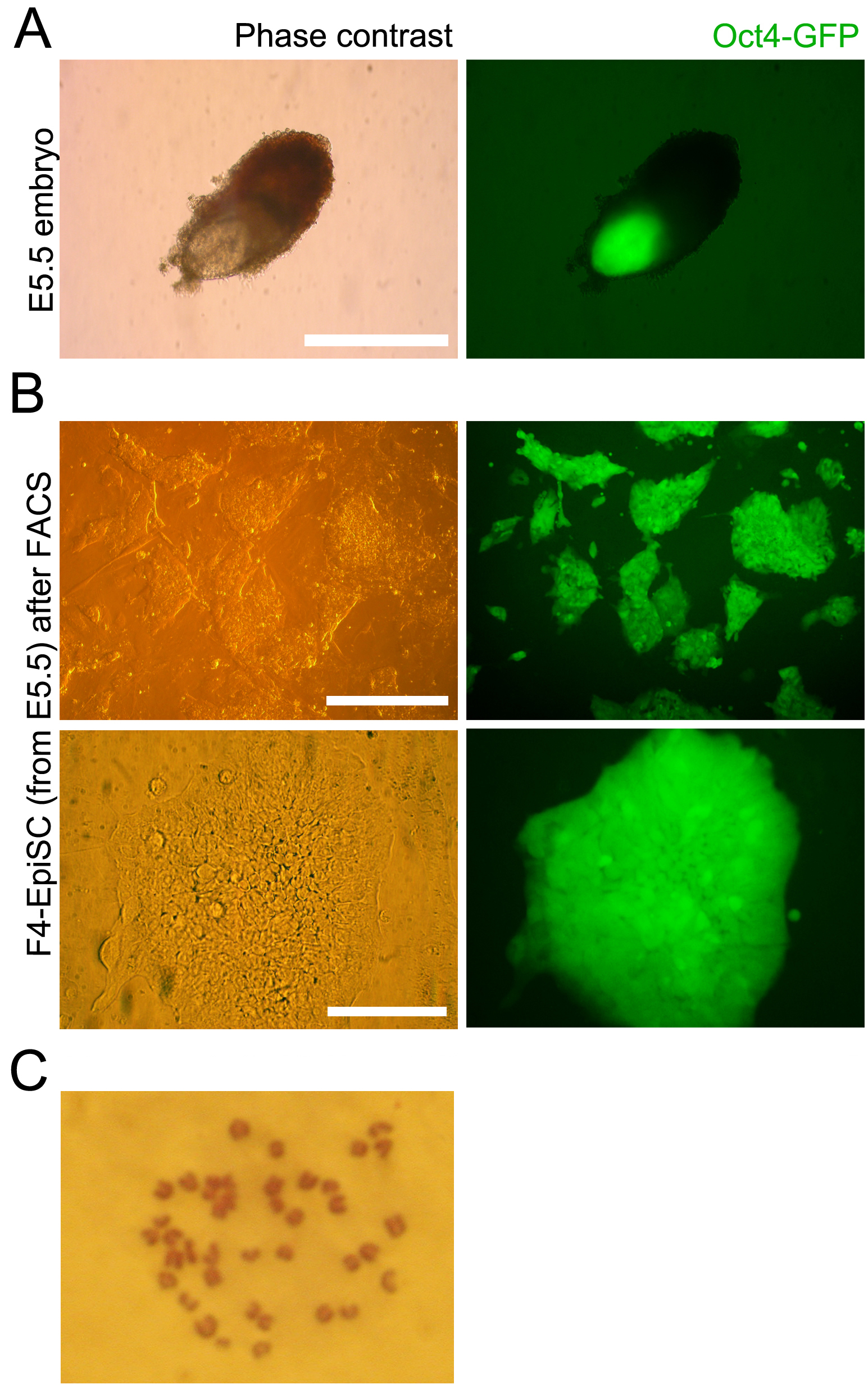
**

**Fig. S1. Generation of EpiSCs from E5.5 epiblast**. (A) E5.5 embryos were recovered from GOF18 transgenic mice. GFP-positive epiblasts were used for generating EpiSCs. (B) Pure populations of GFP-positive cells were established after three rounds of FACS. (C) Homogenous GFP-positive F4-EpiSCs cultured over 100 passages (for about 6 months) maintain normal karyotype. Scale bars are 100 μm.

**
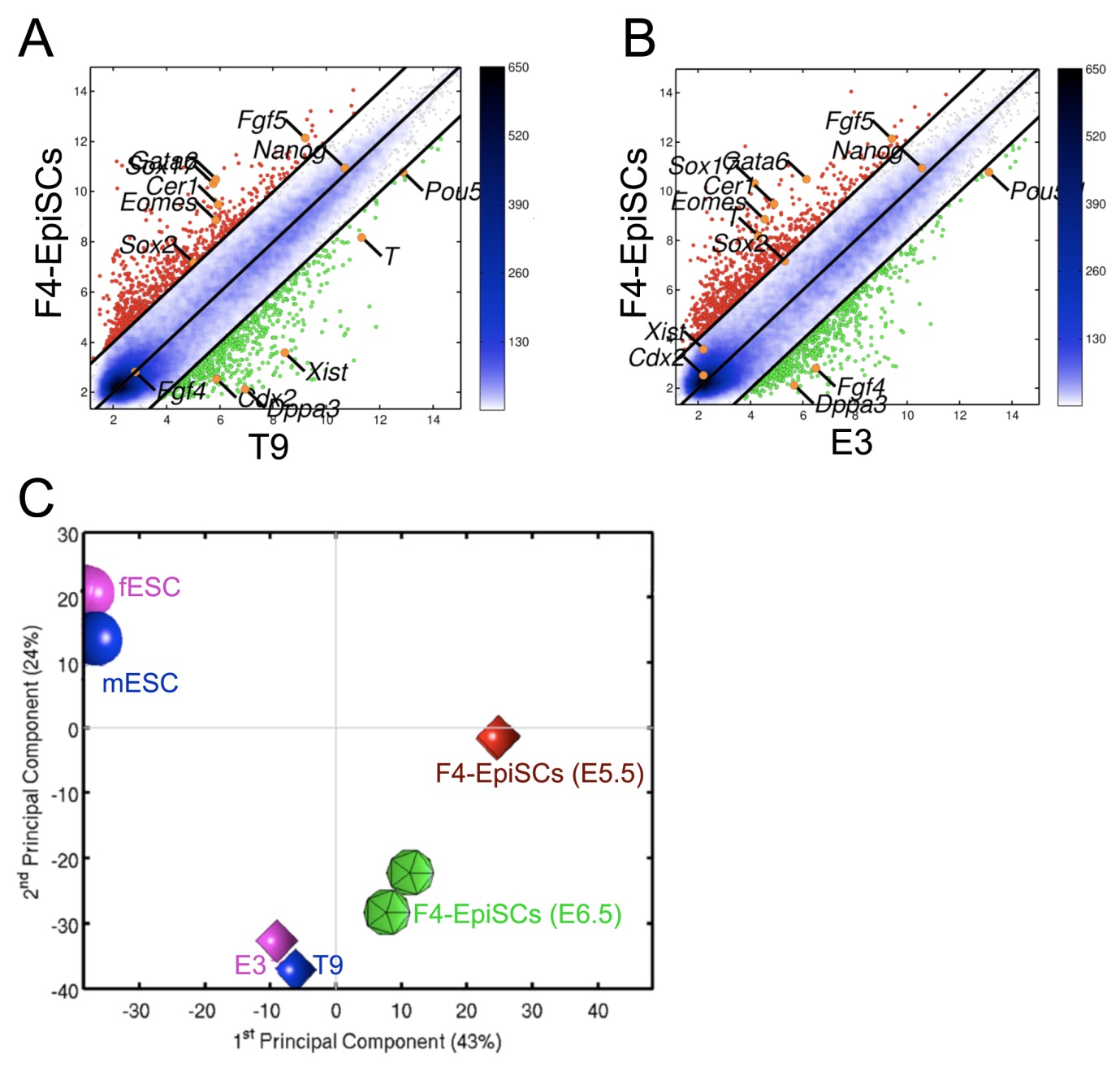
**

**Fig. S2. Scatter plots of global gene expression microarray.** (A) Pairwise scatter plots comparing the global gene expression patterns between F4-EpiSCs and T9 EpiSC line, and (B) between F4-EpiSCs and T9 EpiSC line. Pluripotency (Pou5f1/Oct4, Sox2, and Nanog), germ cell (Dppa3/Stella), and epiblast (T, Fgf5, Eomes, Gata6, and Sox17) markers are indicated with orange dots. The black lines delineate the boundaries of two-fold difference in gene expression levels. The color bar to the right indicates the scattering density. Gene expression levels are depicted on log_2_ scale. (C) Principal component analysis indicate that a global gene profile of F4-EpiSCs are distinctive compared to other EpiSC lines, E3 and T9 and are far more distinct from ESCs.


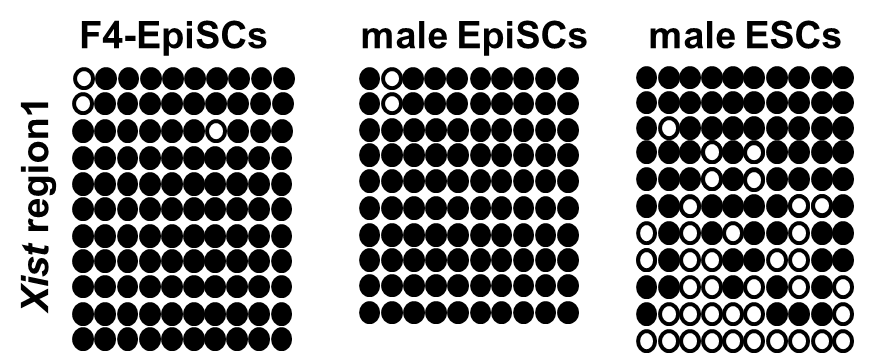


**Fig. S3. Bisulfite DNA sequencing analysis of E6.5 F4-EpiSCs male cell line.** Xist gene region 1 (-381 to +74) of E6.5 F4-EpiSCs was completely methylated, which is the same in the male EpiSC line cultured in conventional medium.
